# A Mobile App (WhiteTeeth) to Promote Good Oral Health Behavior Among Dutch Adolescents with Fixed Orthodontic Appliances: Intervention Mapping Approach

**DOI:** 10.2196/mhealth.9626

**Published:** 2018-08-17

**Authors:** Janneke Francisca Maria Scheerman, Pepijn van Empelen, Cor van Loveren, Berno van Meijel

**Affiliations:** ^1^ Department of Preventive Dentistry Academic Center for Dentistry Amsterdam University of Amsterdam and Vrije Universiteit Amsterdam Amsterdam Netherlands; ^2^ Cluster Oral Hygiene Department of Health, Sports & Welfare Inholland University Amsterdam Netherlands; ^3^ Department of Child Health Netherlands Organization for Applied Scientific Research Leiden Netherlands; ^4^ Cluster Nursing Department of Health, Sports & Welfare Inholland University Amsterdam Netherlands; ^5^ Department of Psychiatry Amsterdam Public Health Research Institute Vrije Universiteit Amsterdam Medical Center Amsterdam Netherlands; ^6^ Parnassia Psychiatric Institute The Hague Netherlands

**Keywords:** health behavior, mHealth, oral health, oral hygiene, dental caries, adolescent, dental plaque, prevention, intervention mapping

## Abstract

**Background:**

The insertion of fixed orthodontic appliances increases the risk of dental caries, particularly in adolescents. Caries can be prevented through good oral health behavior. To support adolescents with fixed orthodontic appliances and for promoting oral health behavior, we developed a theory- and evidence-based mHealth program, the WhiteTeeth app.

**Objective:**

The objective of our paper was to describe the systematic development and content of the WhiteTeeth app.

**Methods:**

For systematic development of the program, we used the intervention mapping (IM) approach. In this paper, we present the results of applying the first 5 steps of IM to the design of an mHealth program: (1) identifying target behaviors and determinants through problem analysis, including a literature search, a survey study, and semistructured interviews, to explore adolescent oral health behavior during orthodontic therapy; (2) defining program outcomes and objectives; (3) selecting theoretical methods and translating them into practical strategies for the program design; (4) producing the program, including a pilot test with 28 adolescents testing the acceptability and usability of the WhiteTeeth app; and (5) planning implementation and adoption.

**Results:**

On the basis of our literature search, we identified fluoride use and control of dental plaque levels (eg, tooth brushing and proxy brush usage) as target behaviors for preventing caries. Next, we identified important and changeable determinants of oral health behavior that fitted the theoretical concepts of the Health Action Process Approach (HAPA) theory. The HAPA theory, the self-regulation theory, and the results of the semistructured interviews were used to define the program objectives, that is, the performance and change objectives. After defining the objectives, we identified multiple behavior change techniques that could be used to achieve these objectives, such as providing oral health information and feedback, prompting self-monitoring, coaching of set actions and coping plans, and sending reminders. We translated these methods into practical strategies, such as videos and a brushing timer. Next, we combined these strategies into a single program resulting in the WhiteTeeth app (which is available on both iTunes and Google Play stores as “Witgebit”). Adolescents with fixed orthodontic appliances and dental professionals were included in the development process to increase the success of implementation. The pilot test revealed that the app users appreciated and liked the app. The WhiteTeeth app can be integrated into current orthodontic care.

**Conclusions:**

IM allowed us to identify multiple techniques that have been shown to be the most effective in initiating behavior change, but have not yet been incorporated into existing orthodontic apps. The WhiteTeeth app contains all these techniques, which makes it a unique and promising home-based app for promoting oral health in adolescents with fixed orthodontic appliances.

**Original Paper**

## Introduction

Dental caries remains a major public health problem that affects young people and adults [1]. Worldwide, nearly 60%-90% of young people and the majority of adults have dental caries, which often leads to pain and discomfort [2-4]. In several industrialized countries, oral diseases are the fourth most expensive disease to treat [2]. Furthermore, 5%-10% of public health expenditure is devoted to oral health treatment [5-6]. Adolescents with fixed orthodontic appliances are at high risk of developing dental caries [7], as fixed orthodontic appliances (eg, brackets) impede oral hygiene procedures, restrict salivary and mucosal self-cleaning capacity, unfavorably altering the balance of oral bacteria and increasing the retention of dental plaque [8-10]. Prolonged dental plaque accumulation can lead to enamel demineralization, which is an early stage of dental caries. Due to their white appearance, these demineralizations are named as *white spot lesions*, which are a common complication in orthodontics [11]. The incidence of patients who develop at least one new white spot lesion during orthodontic treatment ranges from 68% to 95% [12-14]. White spot lesions may develop around the bracket, thus seriously compromising aesthetics [15,16]. After the removal of fixed orthodontic appliances, white spot lesions often remain permanently visible; along with being unaesthetic, they increase the risk of lesion progression [15,16]. Oral health education is essential for the prevention of dental caries in patients with fixed orthodontic appliances. A central role in such education—which is given both before and during orthodontic treatment [17]—involves oral health behavior that targets dental plaque control, dietary behaviors, and fluoride administration [18-21]. However, it is not always easy to achieve regular patient compliance with such oral health behavior [22]. A recent study among Dutch adolescents with fixed orthodontic appliances showed that they had poor overall oral hygiene and poor compliance with the use of fluoride mouth rinse [23]. This emphasizes the need for interventions that focus on changing the oral health behavior in this age group. As growing numbers of young people now have mobile phones, mobile phone apps may be effective means of promoting oral health behavior in orthodontics [24-26]. As a delivery method, apps have many advantages: they are constantly accessible, can be adjusted to the needs of the user, can provide tailored feedback, are more anonymous than face-to-face contact, can send cues to action (ie, reminders), and have a wide reach and interactive features such as animations [27-29]. To promote good oral health behavior among adolescents with fixed orthodontic appliances, we decided to develop a mobile phone app, the WhiteTeeth app (Dutch name: WitGebit app). To ensure that this app would be both theory- and evidence-based and also be feasible for use in orthodontic clinics, we used the IM protocol [30] for its systematic development. This paper provides a detailed description of the development and content of the WhiteTeeth app.

## Methods

### Intervention Mapping Protocol

IM is a protocol for planning and developing theory- and evidence-based health promotion programs [30]. The IM process comprises 6 steps: Step 1, identifying target behaviors and determinants through problem analysis; Step 2, specifying program outcomes and objectives; Step 3, selecting theoretical methods and practical strategies for the program design; Step 4, producing the program; Step 5, planning the implementation and adoption; and Step 6, planning for evaluation [30]. Each step has a defined end product and consists of various tasks that are required for the systematic integration of theoretical and empirical information. The product of a preceding task or tasks guides the developmental activities for the subsequent step or steps.

To guide the developmental process for this intervention, we established a multidisciplinary planning group consisting of an orthodontist, a dental hygienist, two dentists, a mobile phone app developer, a health psychologist, two health scientists, and a child psychologist with communication expertise.

### Step 1: Problem Analysis

The first step of the IM process was to conduct a problem analysis, which included the identification of determinants related to the problem and specific health-related behaviors. The IM process is based on the assumption that health outcomes can be improved by targeting health behaviors and their determinants [30].

To explore the oral health behavior of adolescents during treatment with fixed orthodontic appliances, we conducted semistructured interviews with adolescents with orthodontic fixed appliances (n=20), asking them about their oral health behavior. These semistructured interviews were performed after a regular orthodontic check-up in a private room at the Academic Centre for Dentistry Amsterdam (ACTA).

Adolescents with fixed orthodontic appliances were purposively sampled to ensure that the patient group exhibited a range of sexes, educational levels, ethnicities, and dental hygiene levels. The clinicians told adolescents about the aim of the study and the voluntary nature of participation. Their parents or legal representative were given written information about the study. Informed consent was obtained from both adolescents and their parents.

During the interview, we asked the adolescents about their beliefs and motivations concerning the performance of oral health behavior during fixed orthodontic treatment. Interview topics relevant to the adolescents’ oral health behavior consisted of (1) oral hygiene practices; (2) reasons or motives for performing oral health behavior; (3) awareness and knowledge of dental health and recommendations on oral health (see Textbox 1); (4) personal strategies and reported barriers; (4) role of the social environment; and (5) facilities (accessibility). The adolescents were individually interviewed using open-ended questions to guide the interview. The audiotaped interviews were anonymously transcribed verbatim and transported to a software program “NVivo” to analyze the transcripts. After 20 interviews, saturation was attained, that is, no new relevant information emerged in subsequent interviews. The Medical Ethics Committee of the University of Amsterdam approved this qualitative study (VUMC - 2014-577).

After exploring adolescent oral health behavior during orthodontic treatment through these semistructured interviews, we searched the literature to identify behavioral determinants and theoretical constructs to explain this behavior. We therefore conducted a systematic literature review with a meta-analysis [33]. Since the findings of this review applied to young people in general, not specifically those with fixed orthodontic appliances, we conducted a survey among adolescents undergoing fixed orthodontic therapy (n=116) [23]. This survey study aimed to explain oral health behavior and the presence of dental plaque during orthodontic treatment. A sample of 116 adolescents (12-15 years) with fixed orthodontic appliances was recruited from an orthodontic clinic situated in Almere (Netherlands), and the respondents completed a questionnaire to map their oral health behavior. In addition, a dental hygienist measured their dental plaque levels. Linear regression analyses were performed to examine the factors associated with dental plaque and specific oral health behavior [23].

Next, the planning group selected important and changeable determinants of oral health behavior. According to IM, the importance of determinants is related to their relationships with oral health behavior. The changeability of the determinants that can be achieved by an intervention and the importance of the determinants were established by the development group on the basis of the available scientific literature [23,30,33-40] and consensus judgments.

### Step 2: Identification of Program Outcomes and Objectives

Step 2 involved a detailed specification of program outcomes and objectives indicating those behaviors that needed to change to achieve the overall goal of the program, that is, to prevent dental caries in adolescents during orthodontic treatment, and to prevent existing dental caries from getting worse. The performance objectives formalized the behavioral changes that adolescents with fixed orthodontic appliances needed to make to achieve the behavioral goals of the program (the program outcomes). Per program outcome, two researchers (JS and PvE) defined performance objectives on the basis of the following question: “To perform the desired behavior, what, in concrete terms, do participants in this program need to do?” Next, the same two researchers identified specific determinants that would be deemed useful in changing each performance objective. For example, if a performance objective was “to decide to prevent dental diseases and to change their tooth brushing behavior,” appropriate behavioral determinants may be “risk perception,” “knowledge,” “outcome expectancies,” and “self-efficacy.” Subsequently, we formulated change objectives that stated what determinants needed to change to achieve the performance objectives. The change objectives were the result of combining performance objectives with the changeable determinants of oral health behavior. Thus, to give an example, the determinant was adolescents’ “self-efficacy,” and the performance objective was “adolescents decide to prevent dental diseases and to change their tooth brushing behavior.” In this example, the change objective would be for “adolescents with fixed orthodontic appliances (to) feel able to prevent dental diseases and (to) gain confidence in their ability to brush their teeth twice daily according to the 5-step method.” The first author constructed a matrix, as explained by IM [30], specifying performance objectives, behavioral determinants, and change objectives, which were subsequently validated by the planning group.

### Step 3: Selecting Theoretical Methods and Practical Strategies for Program Design

The third step of IM comprised two phases. In the first phase, we identified and selected theoretical methods. Theoretical methods or behavior change techniques are general techniques or processes that have been shown to enable change in one or more behavioral determinants and which have their origins in behavioral and social sciences theories. One example of a theoretical method is modeling, which is frequently used to facilitate behavior change [30].

**Oral health recommendations for patients with fixed orthodontic appliances from the Academic Centre for Dentistry Amsterdam.**

- To control dental plaque levels, it is recommended to brush teeth at least twice a day according to the “5-step method” and to use dental aids (such as a proxy brush to clean the tooth surfaces around the brackets and to maintain gingival health). The “5-step method” consists of brushing (1) the gingival sites, (2) mesial and distal sites, and (3) incisal sites of the teeth in relation to the position of the bracket on the buccal sites of the teeth; (4) the occlusal sites (chewing surfaces); and (5) the lingual or palatal sites of the teeth. This 5-step procedure takes approximately 3 minutes to fulfill [31].
- Daily use of fluoride mouth rinse and toothpaste during orthodontic treatment is strongly advised for the prevention of dental caries [20,31] .
- Consumption of sugars, refined carbohydrates, and acid or soft drinks should be limited [32].

For each behavioral determinant and in conjunction with the change objective, two researchers (JS and PvE) selected theoretical methods on the basis of the literature on existing dental and orthodontic health promotion interventions [36-52] and behavior change techniques [30,53,54]. For example, to reach the change objective “adolescents monitor their tooth brushing behavior and dental plaque levels,” we selected the methods “self-monitoring of behavior” and “self-monitoring of the outcome of behavior” for changing the determinant “action control.”

In the second phase, we assessed the conditions under which the methods were shown to be effective and translated the selected methods into practical strategies. A practical strategy is “a specific application of a theoretical method, adjusted to the intervention setting, tailored to the target population, and applied considering parameters for effective use of the methods” [30]. For example, the selected method “self-monitoring of behavior” was translated into the practical strategy “adolescents enter into the app whether or not they accomplish their daily oral health tasks.” The planning group decided if the methods and strategies were suitable for the target population and appropriate for designing a mobile phone app. When necessary, small changes were made, resulting in strategies that were easier to implement.

### Step 4: Program Production

In the fourth step, we combined the chosen strategies into a coherent program leading to the development of the WhiteTeeth app. First, the strategies were clustered to create a program plan, which described the intervention components and presented the wireframe drafts. To ensure that the program met the users’ needs and expectations, we organized meetings with the target audience to obtain feedback on the program plan. Helen Parkhurst, a high school in Almere, The Netherlands, allowed us to organize 2 meetings with 30 adolescents (most had current or previous orthodontic appliances) attending preuniversity technology classes.

The first author showed the wireframe drafts and offered a brief demonstration of the main functionalities of the app. As an assignment for a technology class, adolescents were asked to give feedback on the program plan and to design an app. During the second meeting, adolescents presented their app design. New ideas or suggestions for improvements to optimize the program plan were discussed with the planning group. Based on the adjusted version of the program plan, the first author created an adapted version of the app wireframes to increase the app’s acceptability and usability. These adapted wireframes were then improved by a user experience designer. The WhiteTeeth app was developed by ACTA in collaboration with Inholland University of Applied Sciences and TNO Research group. A programmer at ACTA programmed the WhiteTeeth app using Ionic software (Ionicframework.com), which enabled the app to function on two operating systems: IOS≥7 and Android ≥4.1.

To identify aspects of the program that could be improved, the WhiteTeeth app 1.0 was pilot tested. It was first tested for bugs (ie, system errors) by the planning group (resulting in WhiteTeeth 1.1). Second, to increase the app’s acceptability and usability, it was pilot tested for 2 weeks by 28 adolescents with fixed orthodontic appliances, who then provided feedback on its acceptability and usability in an online survey containing 49 questions. The survey measured perceived usefulness, attractiveness, and ease of use and included the System Usability Scale (SUS) for measuring the app’s usability [55]. The SUS scale ranged from 0 to 100 with response ranges from strongly agree to strongly disagree. A SUS score of >68 would be considered above average. This questionnaire has been published elsewhere [56]. The results of the pilot test were used to refine the WhiteTeeth app (resulting in WhiteTeeth 1.2).

### Step 5: Program Implementation Plan

The previous IM steps focused on ensuring the effectiveness of the program. The purpose of the penultimate step of IM is to ensure that the program reaches the intended population by preparing for the adoption and implementation [30]. The planning began by identifying who would use the program, who would adopt it, who would implement it, and who would be responsible for sustaining the program over time. The best way to increase the chances for successful implementation is collaborating with future program implementers from the start of the planning process, thereby linking program developers with program implementers. Dental health professionals were therefore involved throughout the entire process. The planning group discussed the adoption and implementation of the app.

### Step 6: Evaluation Plan

In the final step of IM, an evaluation plan was created. As this final IM step is not within the scope of this paper, it is reported in detail elsewhere [56].

## Results

### Step 1: Problem Analysis

Semistructured interviews with adolescents with fixed orthodontic appliances provided insight into their oral health behavior. These interviews revealed that recommended dental aids, such as proxy brushes, were used only occasionally. Although most respondents stated that they brushed their teeth twice a day as a matter of routine, they often failed to brush for as long as recommended. These respondents had little awareness of the benefit of fluoride, and fluoride mouth rinses were not a preventive measure they chose consciously. The dietary recommendations were familiar to most respondents, but many of them did not fully adhere to these recommendations. The main reasons for performing desired or undesired oral health behavior are listed in Table 1. Respondents felt their parents (especially mothers) were helpful with dental care since they influenced the availability of dental aids and supported the adolescents by reminding them to clean their teeth.

**Table 1:** Main reasons or motives for performing desired or undesired oral health behavior during fixed orthodontic therapy.

Oral health behavior	Reasons for performing or not performing the oral health behavior
Brushing as recommended	Personal appearance and attractiveness (white teeth without discoloration and bad breath)
Not brushing as recommended	Lack of time, forgetfulness, no prioritization, and tiredness
Using dental aids	The necessity they perceived for removing food residues between the brackets
Not using dental aids	They believed that it was unnecessary to follow recommendations with respect to use of these aids: in their view, some dental aids had the same function as the toothbrush. Forgetfulness and uncertainty about their ability to use them correctly.
Rinsing with fluoride mouth rinse	Freshness of breath, better oral health, perceived attractiveness to others due to fresh breath and cleanliness
Not using fluoride mouth rinse	Forgetfulness, not being familiar with the guidelines, or unavailability of mouth rinse at home
Following dietary recommendations	Oral health reasons
Ignoring dietary recommendations	Dietary habits among young people, and social pressure from friends Misperceptions about the recommendations—eg, perceptions regarding the negative effects of soft drinks

The relevant literature was systematically reviewed to identify those behavioral determinants and theoretical constructs that best explained adolescent oral health behavior. The results of this systematic literature review with meta-analysis revealed that the psychosocial factors most strongly correlated with oral health behavior were “self-efficacy,” “intention,” “social influences,” “coping planning,” and “action planning.” These factors are part of the Health Action Process Approach (HAPA) theory [33]. The findings of this review applied to the oral health behavior of young people in general.

Our survey study (n=116) revealed that the HAPA theory could be applied to explain the differences in oral health behaviors in adolescents with fixed orthodontic appliances [23]. According to this theory, behaviors are established in two subsequent phases: (1) a motivational, intention-forming phase and (2) a volitional phase in which intention is translated into action [57]. Regarding the motivational phase, the motivation (ie, intention) to adopt health behaviors is formed by a growing “risk perception,” “outcome expectancies,” and “action self-efficacy.” A minimum level of threat must exist (“risk perception”) before people start considering the benefits of possible actions (“outcome expectancies”) and think about their competence to actually perform these actions (“action self-efficacy”) [57]. Once intentions are formed, the volitional phase starts. The behavioral intention has to be transformed into specific planning of when, where, and how to perform the desired action (“action planning”) and planning of anticipated barriers and ways to overcome them (“coping planning”). Planning is strongly influenced by self-efficacy because self-efficacious individuals achieve mastery through planning, and they visualize successful scenarios that may guide goal attainment (“maintenance or coping self-efficacy”). Persons with confidence in their ability to cope with setbacks will quickly recover when running into unforeseen difficulties (“recovery self-efficacy”). When the behavior has been initiated, self-regulatory cognitions to control and maintain the behavior must be activated (“action control”) [57]. Next, the planning group selected important and changeable determinants of oral health behavior, which are presented in Table 2.

### Step 2: Identification of Program Outcomes and Objectives

The results of the problem analysis were used to specify the program outcomes, performance objectives, and change objectives, which are described below. The program outcomes were specified as follows: (1) Adolescents control their dental plaque levels by improving: (a) their tooth brushing frequency and duration, that is, by brushing their teeth consistently and correctly (5-step method, see Textbox 1) at least twice daily and (b) cleaning around the brackets with a dental aid (eg, a proxy brush). (2): Adolescents increase their exposure to fluoride (ie, a fluoride mouth rinse).

The next stage was to stipulate the performance and change objectives for each of the specific program outcomes. The results of the semistructured interviews (see Step 1), in combination with the frameworks of the HAPA [57] and self-regulatory theory [58] were used to define the performance objectives. Self-regulation theory provides an understanding of the behavioral processes needed for adequate self-management in order to obtain a behavioral goal. As such, it is very useful to define subsets of behaviors. Once the performance objectives had been specified, we created a matrix of change objectives by linking performance objectives to behavioral determinants. In order to design the program, 21 performance objectives and 69 accompanying change objectives were defined. Due to the similarities between the performance objectives for all program outcomes, a selection is presented in Table 3. Table 3 presents 7 performance objectives (PO1-PO7) and 23 change objectives (CO1-CO23) pertaining to program outcome 1a “Adolescents control their dental plaque levels by improving tooth brushing.”

### Step 3: Selecting Theoretical Methods and Practical Strategies for Program Design

After careful consideration of parameters for use, theoretical methods and practical strategies addressing the determinants were selected to achieve the change objectives. The determinants and change objectives, their linked theoretical methods and practical strategies for program outcome 1, “Adolescents control their dental plaque levels by improving their tooth brushing frequency and duration,” are presented in Multimedia Appendix 1. The following paragraphs present the selected theoretical methods and their translation into practical strategies for the same 7 performance objectives (PO; Step 2).

#### Performance Objective 1—Providing Health Risk Information, Personal Advice, and Instructions

Suitable methods for supporting decision-making on oral health behavior include providing health risk information on oral health behavior and giving personal advice and instructions (targeting determinants: “risk perception,” “outcome expectancies,” and “knowledge”) [53]. To personalize dental advice and instructions, the app collects information on adolescents’ oral health behavior and dental plaque levels. Adolescents were asked to answer questions covering their tooth brushing frequency, their use of fluoride mouth rinse and dental cleaning aids, the duration of their brushing sessions, and the type of toothbrush they used. Next, they were asked to use disclosing tablets in order to visualize their dental plaque. The app then showed an example of a selfie, asked them to take a selfie of the teeth where plaque was visualized, and also asked them to indicate the plaque by clicking on the selfie (the app is installed in the orthodontic clinic, where a dental hygienist provided instructions on using the disclosing tablets and using the mobile phone to take a selfie of the teeth). Based on the number of clicks (ie, the amount of plaque) and answers to the questions, the app provided personal advice on oral health behavior (see Table 4 for the algorithm). If an adolescent did not adequately control his or her plaque levels or if his or her oral health behavior was poor, health risk information was offered via a short animated movie, which depicted the likely development of white spot lesions. This and an image of beautiful white teeth were shown as outcomes resulting from complying with oral health recommendations and thus provided adolescents with motivation for performing the desired oral health behavior.

**Table 2:** Selection of significant determinants of oral health behavior^a^.

Determinants			Importance^b^		Changeability^c^		Evidence for importance	
**Personal**								
	Knowledge and awareness			+^d^		+++		r=0.20; *P*<.001
	Risk perception			+		+		Precondition for personal relevance
	Attitude and expectancies			++^e^		+		r=0.20; *P*<.001
	Subjective norm			++		+		r=0.26; *P*<.001
	Self-efficacy			+++^f^		+		r=0.37; *P*<.001
	Intention			+++		+		r=0.40; *P*<.001
	Planning (action and coping)			+++		+		r=0.52; *P*<.001
	Self-regulatory skills, such as action control and goal commitment			+++		+		Maintaining behavior
	Motor skills			++		+		Precondition for improvement in self-efficacy
	Habit			+++		+		Making a certain behavior automatic
**External**								
	**Social influences**							
		Parental behavior		+++		+		r=0.4; *P*<.001
		Dental professional		+		+		Based on consensus judgments of the development group
	Cues			+++		+		Most direct environmental influence
	Access or Availability			+++		+		Making healthy behavior easier

^a^Correlation and significant levels are based on results from previous studies on oral health and behavior change [23,30,33-40].

^b^Importance: the strength of the evidence for the relationship between the determinant and oral health behavior we want to change.

^c^Changeability: the strength of the evidence that the proposed change can be realized by a program.

^d^+: not very important, not easy to change.

^e^++: important, changeable.

^f^+++: very important or easy to change.

**Table 3:** Seven performance objectives (PO1-PO7) and 23 change objectives (CO1-CO23) pertaining to program outcome 1a “Adolescents control their dental plaque levels by improving tooth brushing.”

Performance objective and determinant		Change objectives
**PO1: Adolescents decide to prevent dental diseases and to change their tooth brushing behavior**		
	Risk perception	CO1: Are aware of their susceptibility to dental diseases
	Awareness	CO2: Are able to describe their tooth brushing behavior
	Knowledge	CO3: Know what good oral health is and its association with dental plaque
	Risk perception, Expectancies	CO4: Acknowledge the risk of not brushing teeth as recommended and its consequences
	Expectancies	CO5: Know the benefits of maintaining good oral health
	Knowledge	CO6: Know how to brush teeth according to the 5-step method
	Self-efficacy	CO7: Feel able to prevent dental diseases and gain confidence in ability to brush teeth twice daily according to the 5-step method
	Skills	CO8: Develop tooth brushing skills (5-step method) to remove all dental plaque
**PO2: Adolescents choose or plan how to improve their tooth brushing behavior**		
	Goal commitment, Self-efficacy	CO9: Choose a change about which they feel self-efficacious
	Skills	CO10: State a clear tooth brushing or oral hygiene goal
**PO3: Adolescents prepare strategies to establish how they will change their tooth brushing behavior.**		
	Action planning	CO11: Plan in terms of when and where to brush their teeth
	Attitude	CO12: Show commitment to their goals
**PO4: Adolescents change their tooth brushing behavior**		
	Support	CO13: Receive support during brushing on where and for how long to brush teeth
	Cues to action	CO14: Receive cues to tooth brushing
**PO5: Adolescents evaluate their tooth brushing behavior, their dental plaque levels, and the effect of brushing on these levels**		
	Self-regulatory skills—action control	CO15: Monitor their tooth brushing behavior and dental plaque levels
	Self-regulatory skills, Awareness	CO16-17: Examine how well their performance corresponds to agreed goals, and consider modifying goals accordingly
**PO6: If adolescents have difficulty attaining their tooth brushing or dental plaque goal, adolescents identify possible solutions**		
	Coping planning, Action control	CO18: Identify and anticipate barriers and ways to overcome them
	Self-efficacy	CO19: Gain confidence to deal with possible barriers
	Social influences	CO20: Enlist others to help overcome barriers
**PO7: Adolescents maintain the desired tooth brushing behavior**		
	Self-efficacy	CO21: Gain confidence in maintaining tooth brushing behavior
	Expectancies	CO22: Feel positive about tooth brushing
	Attitude	CO23: Believe that long-term benefits can be achieved by maintaining tooth brushing over time

**Figure 1:**
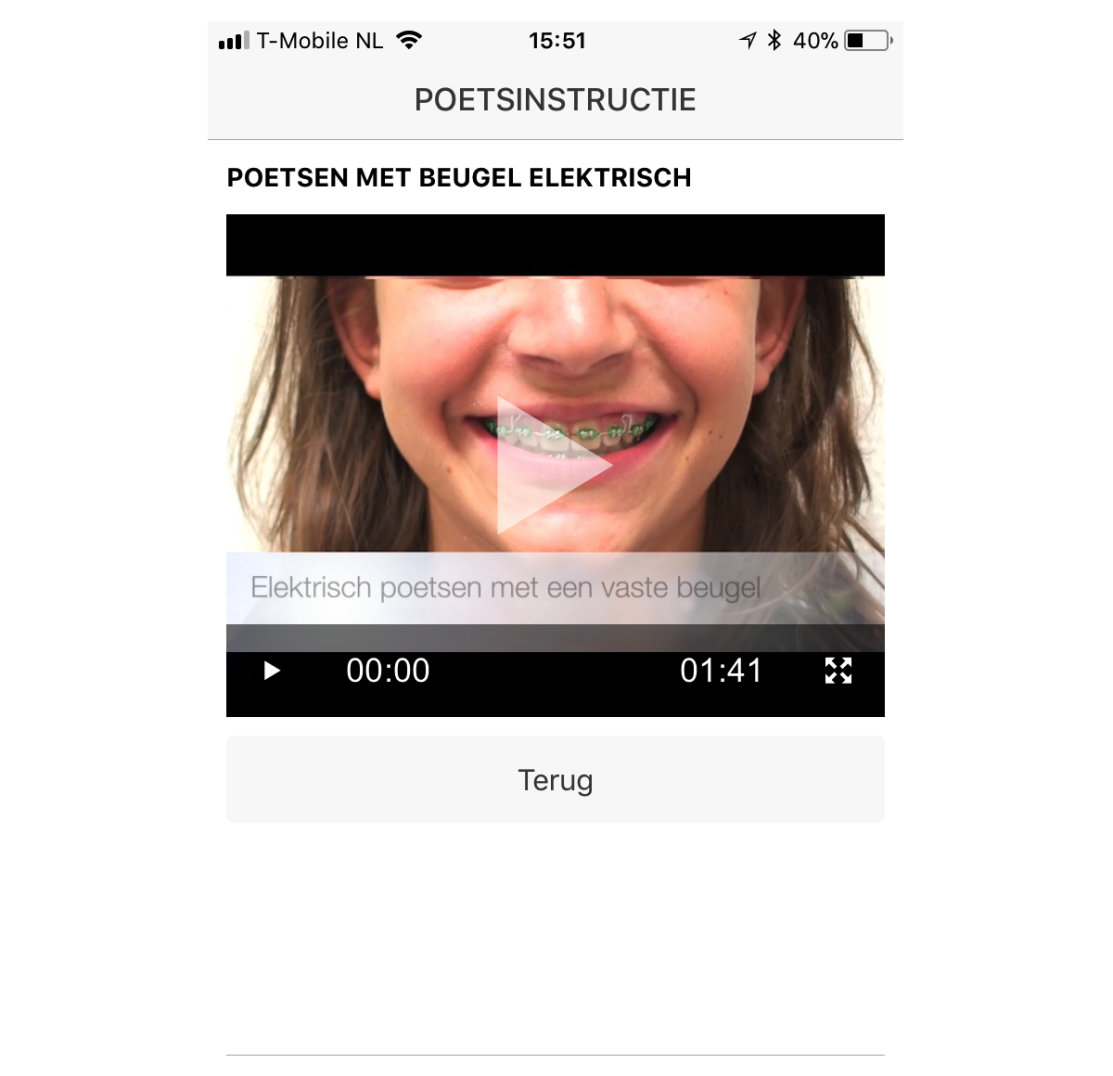
Screenshot of a movie of the WhiteTeeth app. Taken on an iPhone, this movie shows users a peer model who demonstrates how to use an electric toothbrush to brush teeth fitted with fixed orthodontic appliances.

Our semistructured interviews showed that doubts about personal oral hygiene skills and the perceived complexity of the techniques were important barriers to the use of dental cleaning aids. To target adolescents’ self-efficacy, movies of a peer model were shown (adolescent with fixed orthodontic appliances), demonstrating how to clean teeth correctly (according the 5-step method—see Textbox 1) that have fixed orthodontic appliances (Figure 1) [59]. This demonstration was tailored to the kinds of toothbrushes the adolescents used.

#### Performance Objective 2—Goal Setting

Goal setting can help adolescents to choose how to improve their oral health behavior (targeting the determinant: “skills”) [34]. Important conditions for the success of goal setting are the adolescent’s commitment to the goal and the fact that the goals are challenging, but lie within the adolescent’s abilities to achieve them. To ensure their commitment, adolescents chose a health behavior goal that best matched their preferences and abilities. In a series of questions, the app guided them through the process of defining one or more oral health goals. The adolescents then selected an oral health behavior he or she would like to change, for example, improving the frequency and duration of tooth brushing and the use of a proxy brush or a fluoridated mouth rinse. The answers were presented as clear goals on the main page of the app.

#### Performance Objective 3— Planning and Behavioral Contracting

Planning (ie, formulating action plans) and contracting were identified as methods for preparing oral health behavior change (targeting determinants: “action planning” and “attitude”) [37-39,44-46]. The app asked questions, which guided the adolescents in the creation of action plans by specifying goals, in terms of when and where they should act. The answers were presented as their action plan, which would state where and when they would brush their teeth. This action plan was formulated as an implementation intention (“If situation X arises, then I’ll do Y”). When one or more goals were formulated, the adolescent agreed to the overall action plan by signing a contract in the app. This was saved on its main page. The action plan was linked to the option for setting reminders.

#### Performance Objective 4—Practical Support (the Brushing Timer)

**Figure 2:**
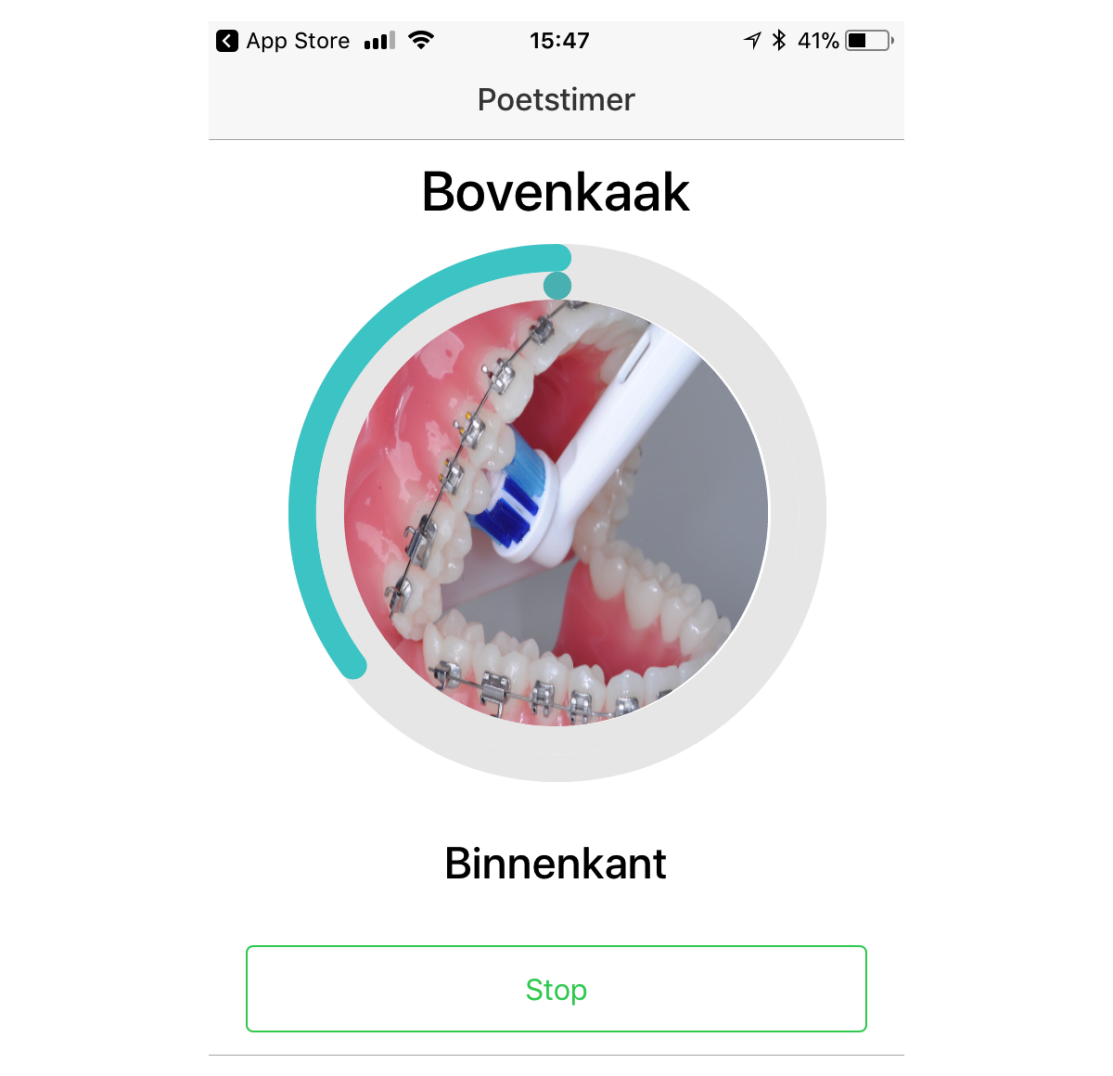
Screenshot of the brushing timer. Taken on an iPhone, it shows how adolescents can use the brushing timer (poetstimer) to see how much time has elapsed and also where to brush; in this case, the inside (binnenkant) of the maxilla (bovenkaak).

To establish oral health behavior change, practical support was identified as a useful method [53]. To provide practical support, the app incorporated a brushing timer, which users could turn on when they decided to brush (targeting determinants: “support”). The timer showed how much time had elapsed. Throughout brushing, it also supported good brushing, according to the 5-step method, by showing where to brush (location in the mouth). Figure 2 shows a screenshot of the brushing timer. When brushing with the brushing timer was completed, the app congratulated the user on fulfilling the task.

**Table 4:** The algorithm for personal recommendations that were provided based on plaque assessment and answers to the registration questions.

Flow	Answer options (the answer)	Interpretation of the answers and personal recommendations
1.1	Question A: Tooth brushing frequency <2 times/day (0/1), OR Question B: Tooth brushing duration <3 min/day (0/1/2), OR Dental plaque is visible on the selfie.	The user does not follow the tooth brushing recommendations and dental plaque is present. The app provides information on health risk plus recommendations and instructions. It helps to set goals for increasing brushing frequency and duration. It advises users to use the brushing timer and to monitor their tooth brushing frequency daily.
1.2	Question A: Tooth brushing frequency >2 times/day (2/3 or more often), AND Question B: Tooth brushing duration ≥3 min/day (3/4 min or longer), AND Dental plaque is or is not visible on the selfie.	The user follows the tooth brushing recommendations and dental plaque is absent or present. Continue to question C—flow 2.
2.1	Question C: Proxy brush usage <1 time/day (0), OR Dental plaque is or is not visible on the selfie.	The user does not follow the proxy brush recommendations or dental plaque is present. The app provides information on health risk plus recommendations and instructions. It helps to set goals for increasing the use of a proxy brush and for increasing tooth brushing frequency and duration. It advises users to use the brushing timer and to monitor their tooth brushing frequency and proxy brush usage.
2.2	Question C: Proxy brush usage 1 time/day (1/2 or more often), AND Dental plaque is visible on the selfie.	The user follows the proxy brush recommendations, but dental plaque is present. Idem as flow 2.1.
2.3	Question C: Proxy brush usage 1 time/day (1/2 or more often), AND Dental plaque is not visible on the selfie.	The user follows the proxy brush recommendations and dental plaque is absent. Continue to question D—flow 3
3.1	The user does not have 3 fluoride moments per day: Question A: Tooth brushing frequency <3 times/day (0/1/2), OR Question D: Fluoride mouth rinse usage <1 time/per day.	The user does not follow the fluoride recommendations. The app provides information on health risk plus recommendations and instructions. It helps to set goals for increasing the use of fluoride mouth rinse. It advises users to monitor their fluoride mouth rinse usage.
3.2	The user has 3 fluoride moments per day: Question A: Tooth brushing frequency ≥3 times/day (3 or more often), OR Question D: Daily fluoride mouth rinse usage.	The user follows all recommendations. Positive reinforcement.

#### Performance Objective 4— Prompt Cues (Reminders)

Since numerous studies have shown that sending short message service (SMS) text messages as prompt cues is an effective way for establishing behavior changes and improving oral hygiene during fixed orthodontic treatment [48-50], the app also provided an option for setting reminders for oral health behavior tasks (including monitoring of behavior and dental plaque) and the use of the brushing timer (targeting determinants: “cues to action or habit formation”). The reminders were sent as push notifications.

#### Performance Objective 5—Prompt Self-Monitoring

We identified prompt self-monitoring as a suitable method for evaluating tooth brushing behaviors and dental plaque levels (targeting determinants: “self-regulatory skills or action control” and “awareness”) [36,40,51,52,60]. The use of disclosing agents provided a suitable method of monitoring plaque levels and thereby improved oral hygiene [60]. When the app was installed in the orthodontic clinic, a dental hygienist explained how oral health behavior and plaque levels should be monitored. The next day, the app sent a push notification that urged the adolescents to monitor their oral health behavior daily by entering into the app whether they accomplished their daily dental activities. If they failed to complete the monitoring, a push notification was sent the next day. Each week, adolescents were asked, via the app, to evaluate their dental plaque levels and review their behavioral goals. For this purpose, they were asked to use a disclosing tablet to visualize the dental plaque, to take a selfie of the result, and to indicate the visualized dental plaque. On the basis of the information on the selfie and the activities performed that week, the app concluded whether the adolescent’s goals had been attained. It then congratulated the adolescent for using the app, and if necessary, guided him or her in setting goals or adapting existing ones and in creating coping plans.

#### Performance Objective 6— Prompt Barrier Identification to Establish Coping Plans (Volitional Sheets)

We identified prompt barrier identification and the creation of coping plans as suitable methods for helping adolescents to identify possible ways of achieving their oral health goal if they encountered difficulties (targeting determinants: “self-regulatory skills or action control,” “coping self-efficacy,” and “coping planning”) [40,61,62]. If adolescents failed to attain their goals, coping plans could be formulated [57]. These plans use “if-then” formulations to specify how they would deal with difficult situations. However, although adults realized positive effects for if-then planning (ie, implementation intentions [61-62] on oral health behavior were undertaken with adults [40]), it is possible that planning interventions would be less suitable for adolescents, who may be less familiar with creating behavioral coping plans. To mitigate this, the app therefore incorporated volitional help sheets [63]—a tool for constructing effective (if-then) coping plans—by asking participants to link difficult situations (where “if” indicates barriers against performing the desirable behavior) with a behavioral response (where “then” indicates solutions) [47]. For example, “If I often forget to brush my teeth, then I ask someone at home to remind me to brush my teeth.” Table 5 shows the content of a volitional help sheet intended to establish coping plans for tooth brushing behavior. The content of the volitional help sheets was informed by the results of the semistructured interviews (performed in Step 1). To remind the adolescents of their coping plans, the plans were saved on the main page of the app, and thus were visible when the app was opened.

**Table 5:** An example of the content of the volitional help sheet used to establish coping plans for tooth brushing behavior.

Difficult situations (Think about difficult situations that hinder tooth brushing and possible solutions to them. Please select the difficult situations and solutions that fit you best)	Possible solutions
□ (If) I am too tired to brush my teeth	□ Then I think of the dentist who has to fill all the cavities
□ (If) I don’t feel like tooth brushing	□ Then I think of the brown spots and cavities I might get if I don’t brush my teeth
□ (If) I want to skip tooth brushing because I’m in a hurry	□ Then I think about how fresh and clean my teeth will feel after brushing
□ (If) want to skip tooth brushing because I’ve got something much more fun to do	□Then I ask someone at home to remind me to brush my teeth
□ (If) I often forget to brush my teeth	□ Then I think about what the orthodontist or assistant told me about brushing my teeth
□ (If) I’m so busy that I don’t have time for tooth brushing	□ Then I think about the bad breath I can get if I don’t brush my teeth
□ (If) I prefer not to brush my teeth because they’re sensitive or painful	□ Then I set a reminder
□ (If) I don’t want to brush my teeth because it’s too difficult	□ Then I think of tooth brushing giving me fresh breath and white teeth
□ (If) I prefer not to brush my teeth because my gums are bleeding	□ Then I look in the mirror and say to myself: “I can do it! Every day!”
□ (If) I’m too tired to brush my teeth in the evening	□ Then I watch the movie about tooth brushing in the app
□ (If) I… (option to fill in)	□ Then I’ll brush my teeth right after dinner
	□ Then… (option to fill in)

#### Performance Objective 7—Providing Positive Reinforcement (Coaching Short Message Service Text Messages)

Maintaining oral health behavior requires long-term commitment. Providing reinforcement by sending coaching SMS text messages was identified as a suitable method of motivating adolescents to maintain the desired behavior (targeting determinants: “attitude” and “maintenance self-efficacy”) [53]. To personalize coaching SMS text messages, adolescents were asked what outcomes motivated them to maintain good oral health. They could select from pre-established motives such as “keeping my gums healthy,” “getting fresh breath,” or “white teeth.” If desired, these notifications could be switched off.

### Step 4: Program Production

The practical strategies were clustered into 4 main program components: (1) Registration to help adolescents to decide to change their oral health behavior, to choose how to change it, and to plan appropriate actions; (2) behavior change to help adolescents to actually change their behavior with respect to their daily oral health routines; (3) evaluation to help adolescents to evaluate their behavior change over the past week and to adapt goals weekly; and (4) maintenance to help adolescents to maintain their behavior. Textbox 2 shows an overview of the flow of the program.

#### The Final Program: the Whiteteeth App

The app was listed on both iTunes and Google Play stores as the “WitGebit” app. The WhiteTeeth (“WitGebit”) app was made available free of charge for IOS≥7 and Android≥4.1 operating systems.

#### Pilot Test of the WhiteTeeth App

The most important finding of the pilot test was that adolescents with fixed orthodontic appliances liked and appreciated the WhiteTeeth app, particularly the movies with instructions on how to use proxy brushes. The mean SUS score was 77, indicating an acceptable score for usability. Since the app users suggested changing the amount of storage of the WhiteTeeth app, we compressed the movies to reduce the storage of the app to 52.8MB. The app users also suggested improving the instructions for the brushing timer and the statistics for evaluating their behavior. Even though the users requested gamification, this could not be included due to financial limitations. The program was adapted using their feedback.

### Step 5: Program Implementation Plan

The planning group agreed to deliver the intervention through dental professionals that already had regular contact with adolescents receiving orthodontic therapies, thereby allowing the app to be implemented within existing oral health care processes. One of the barriers to implementation perceived by the dental professionals was the limited time they had during appointments. They therefore recommended that we created an app that could operate as a stand-alone program. To encourage adolescents to use the WhiteTeeth app, several practical strategies were planned. For example, if the adolescents did not use the app for 3 days, the app used the registration information to send personalized SMS text messages reminding them to use the app, such as “Brushing your teeth will help to keep them healthy and beautiful.”

**An overview of the flow of the WhiteTeeth app: targeted performance objectives (POs).**

1. Registration—First day (PO1- PO3)
  - Users are required to respond to registration questions and provide some personal information.
  - The app asks users to visualize dental plaque using disclosing tablets and to indicate the plaque on the selfie.
  - On the basis of the information collected on their oral health behavior and dental plaque, the app then provides health risk information, personal advice and instructions in short videos.
  - Next, it helps the users to customize their personal oral health goals, creating action plans and setting reminders.
  - At the end of installation, it encourages them to use the brushing timer and monitor their oral health behavior every day.
2. Behavior change—Every day (PO4)
  - When they decide to brush, they have the option of turning on the timer. Afterward, the app provides positive reinforcement.
  - Users receive a push notification on a daily basis to monitor their behavior.
3. Evaluation—Every week (PO5, PO6)
  - Users are asked by the app to evaluate their dental plaque levels, to review their behavioral goals, and to create coping plans if needed.
4. Maintenance—Every 3 days (PO7)
  - Users receive coaching short message service text messages.

## Discussion

### Principal Findings

This paper describes the development process and content of the WhiteTeeth app. The WhiteTeeth app was developed to promote oral health behavior among adolescents with fixed orthodontic appliances who were at high risk of developing dental caries. We used an IM protocol as a tool for the systematic development of the app [30]. IM linked the phases of intervention development to theory and empirical evidence and made the process of program development transparent. IM was proven to be a suitable method for developing health promotion programs for various health issues [64-66]. In the field of orthodontics, authors did not describe the process of program development explicitly in their publications [41,42,48-50,67-71]. This limited opportunities for comparison. Mapping the development and contents of an intervention, as in this study, is useful because it allows researchers to faithfully replicate effective programs or design programs that are even more effective [72]. In contrast to other studies, our study used theory to inform the program design. The use of theory was necessary to ensure that the factors related to achieving change were addressed [73,30]. When reviewing the few available orthodontic apps promoting oral health, we concluded that the integration of behavior change techniques was limited in these apps [25,70-71,74-75]. However, a meta-analysis revealed that programs with a larger differentiation of behavior change techniques tended to have larger effects on behavior than programs that incorporated fewer techniques, which may be a consequence of the fact that different techniques target different aspects of the behavior change process [73]. In addition to this matter, behavior change techniques that were most effective for initiating behavior change, such as creating action and coping plans, were not incorporated into these apps [73,76]. Our app contains multiple proven techniques that focus on the motivation and initiation of oral health behavior changes. We believe this makes it a unique and promising mHealth program for oral health promotion. Our work represents a major contribution to the field of oral health care, as it is the first study to systematically develop an mHealth program based on sound evidence and theory. The involvement of dental professionals and adolescents enabled us to develop a feasible program, which offered ample opportunities for effective implementation in the future. To increase the likelihood that the app would meet the preferences of the target group, we invited a user experience designer to participate in the app development and also included future users through semistructured interviews and a pilot test. Interaction with the adolescents enabled us to create program materials, such as volitional sheets that listed barriers and solutions, suited to the individual situations of target group members. Our problem analysis helped us to identify important determinants that were not addressed by the existing oral health programs, such as volitional factors that are outlined in the HAPA theory [57]. Using the IM protocol ensured that all important program objectives were addressed in the WhiteTeeth app, based on the theoretical insights and methods, empirical findings, and practical strategies.

### Limitations

However, there were some limitations that should be highlighted. Despite the value of this robust development process, IM is very time-consuming. Our experience in this regard was similar to that of other researchers who used the IM protocol [77-81]. Our development process required more time than expected because we had to carry out additional research to gain insights into oral health behavior and its determinants during orthodontic treatment (Step 1), as there was little information available on these topics. Another challenge regarding IM, as others have acknowledged [79-82], was the complexity of detailing the performance and change objectives. Program developers and researchers recognized that targeting multiple complex behaviors may create a high degree of complexity since data obtained during the development process can become cumbersome and overwhelming [80,82]. In our study, the creation of matrices of change objectives was particularly time-consuming and resulted in an overwhelming amount of information about what should be targeted by the program. During our development process, we excluded an important target behavior, intake of sugar-sweetened beverages, in order to manage the data of our study and the complexity of our program [83].

### Conclusions

and promising mHealth intervention for adolescents with fixed orthodontic appliances. This app incorporated several behavior change techniques, such as self-monitoring, goal setting, and volitional sheets. The app simultaneously targeted important determinants of oral health behavior change. The lessons learned from using the IM process have relevance for researchers and practitioners, especially considering the current paucity of evidence-based oral health promotion programs for orthodontic patients and their failure to incorporate important behavior change techniques addressing meaningful behavioral determinants. Our future randomized controlled trial will indicate whether the app is effective in improving adolescent oral health.

## Appendix

Multimedia Appendix 1 Performance objectives, selected change objectives, theoretical methods, and practical strategies for program outcome 1 “adolescents control their dental plaque levels by improving their tooth-brushing frequency and duration.”

## Abbreviations

ACTA: Academic Centre for Dentistry Amsterdam
CO: change objective
HAPA: Health Action Process Approach
IM: intervention mapping
PO: performance objective
SUS: System Usability Scale
