# Supplementary material for: A Mobile App (WhiteTeeth) to Promote Good Oral Health Behavior Among Dutch Adolescents with Fixed Orthodontic Appliances: Intervention Mapping Approach
Source: JMIR Mhealth Uhealth. 2018 Aug 17;6(8):e163. doi: 10.2196/mhealth.9626 (PMC6119215; doi:10.2196/mhealth.9626)
Supplement: Multimedia Appendix 1 [file mhealth_v6i8e163_app1.pdf]

MULTIMEDIA APPENDIX A

Table. Performance objectives, selected change objectives, theoretical methods and practical strategies for program outcome 1 “adolescents control their dental plaque levels by improving their tooth-brushing frequency and duration”.

PO1	Adolescents decide to prevent dental diseases and to change their tooth-brushing behavior.			
	Change objectives (determinant)	Theoretical methods/ BCTs *	Requirements	Practical strategies
	Adolescents are aware of their susceptibility to dental diseases (risk-perception).<br><br>Adolescents are able to describe their tooth-brushing behavior (awareness).	- Providing feedback on behavior (BCT 2.2): i.e., monitoring and providing informative or evaluative feedback on performance of behavior [1, 2].<br><br>- Using disclosing tablets to visualize and evaluate dental plaque [3].<br><br>- Providing feedback on outcome (BCT 2.7): i.e., monitoring and providing feedback on the outcome of the behavior [1, 2].	Adolescents need to possess some sub-skills (they need to know how to use the disclosing tablets and take a selfie).<br><br>Feedback needs to be personal and specific.	In the orthodontic clinic (upon installing the app), a dental hygienist demonstrates how to use the disclosing tablets to visualize plaque and how to take a selfie.<br><br>To provide feedback, information on adolescents’ oral health behavior and dental plaque levels will be collected. Adolescents will first be asked to answer to questions with regard to their oral health behavior. Next, adolescents are asked to use disclosing tablets to visualize the dental plaque. After they use the tablets, they are asked to take a selfie of their teeth with the visualized plaque (red color). The app presents an example of a selfie and the selfie will be stored on the main page of the app. The app asks them to click where the plaque is present on the selfie. Based on the number of clicks and registration questions, the app provides feedback on their dental plaque levels and oral health behavior, and provides oral health advice.
	Adolescents know what good oral health is and its association with dental plaque (knowledge).<br><br>Adolescents acknowledge the risk of not brushing teeth as recommended and its consequences (risk-perception & outcome expectancies).<br><br>Adolescents know the benefits of maintaining good oral health (outcome expectancies).	- Providing information on health consequences (BCT 5.1) (e.g. written, verbal, visual): i.e., providing information on health consequences of performing the behavior) [2].<br><br>- Belief selection: i.e., using messages designed to strengthen positive beliefs, weaken negative beliefs, and introduce new beliefs [1].<br><br>- Providing scenario-based risk information: i.e., providing information that may aid the construction of an image of the ways in future loss or accident	There must be a plausible scenario with a cause and an outcome.<br><br>The message must be easy to understand: any use of imagery or movies must be relevant and must not diverge too much from the target group’s experience.	The app provides information on the importance on tooth brushing as recommended in a short animated movie. The movie explains that not brushing teeth can increase the risk of getting dental diseases (how plaque can cause dental diseases) and can also affect appearance. The movie provides information on the positive outcomes of maintaining good oral health. It shows two scenarios: (1) bad oral hygiene causes white spot lesions/dental caries; (2) good oral hygiene causes white and beautiful teeth.

		might occur [1].<br><br>- Providing reinforcement: i.e., linking a behavior to any consequence that increases the rate, frequency and probability of the behavior [1].		
	Adolescents know how to brush teeth according to the 5-step method (knowledge).<br><br>Adolescents feel able to prevent dental diseases and gain confidence in ability to brush teeth twice daily according to the 5-step method (action self-efficacy)<br><br>Adolescents develop tooth-brushing skills (5-step method) to remove all dental plaque (skills).	- Providing instructions on how to perform the behavior (BCT 4.1): advise or agree on how to perform behavior (includes "skills training') [2].<br><br>- Demonstrating the behavior (BCT 6.1): i.e., providing an observable sample of the performance of the behavior, directly in person or indirectly (e.g. through film) (includes "modeling" [1, 2].	The adolescents must identify with the model (use a coping model instead of mastery model).	The app provides a movie in which a peer model (adolescent with fixed orthodontic appliances) demonstrates how to brush teeth correctly twice daily according to the 5-step method. The demonstration is tailored to the kind of toothbrush they use, i.e., electric or manual.
PO2	Adolescents choose/plan how to improve their tooth-brushing behavior.			
	Change objectives (determinant)	Theoretical methods/ BCTs	Theoretical requirements	Practical strategies
	Adolescents choose a change about which they feel self-efficacious (goal-commitment & action self-efficacy).	- Prompting intention formation: i.e., encouraging the person to decide to act or set a general goal [27].<br><br>- Setting graded tasks: i.e., setting easy tasks, and increasing difficulty until target behavior is performed [1].	The target behavior can be reduced to easier but increasingly difficult sub-behaviors.	The app allows the adolescents to choose the tooth-brushing duration and/or frequency they would like to change and they think they can change.
	Adolescents state a clear tooth-brushing or oral hygiene goal (skill).	- Goal-setting (behavior) (BCT 1.1): i.e., setting or agreeing a goal defined in terms of behavior to be achieved [2].		Guided by questions in the app, adolescents set a clear goal. The answers are presented as clear goals, which are stored on the main page of the app.
PO3	Adolescents prepare strategies to establish how they will change their tooth-brushing behavior.			
	Change objectives (determinant)	Theoretical methods/ BCTs	Theoretical requirements	Practical strategies
	Adolescents plan in terms of when and where to brush their teeth (action planning).	- Prompt action planning (BCT 1.4): i.e., prompting detailed planning of performance of the behavior (must include at least one of context, frequency, duration and intensity) [2]. (This	Adolescents need to have a positive intention.	The app incorporates a program that helps the adolescents to form action plans. Guided by questions in the app, adolescents can specify when and where they will brush their teeth. The answers are presented as their action plan, which will state where and when they will brush their teeth. This

		includes implementation intentions: prompting making if-then plans that link situational cues with responses that are effective in attaining goals or desired outcomes [1]).		action plan is formulated as an implementation intention ("If situation X arises, then I'll do Y") (The action plan is linked to the option for setting reminders).
	Adolescents show commitment to their goals (attitude).	- Behavioral contract (BCT 1.8): i.e., creating a written specification of the behavior to be performed, agreed by the person, and witnessed by another [2].		The app get the adolescent to sign a contract with the tooth-brushing goals formulated at a previous stage. This goal (i.e., action plan) will be stored on the app's main page.
PO4	Adolescents change their tooth-brushing behavior.			
	Change objectives (determinant)	Theoretical methods/ BCTs	Theoretical requirements	Practical strategies
	Adolescents receive support during brushing on where and for how long to brush teeth (facilitator/support).	- Providing practical support (BCT 3.2): i.e., advising on, arranging, or providing practical help for performance of the behavior [2].<br><br>- Providing technical assistance: i.e., providing technical means to achieve desired behavior [1].	The technical assistance must fit the recipient's needs and resources.	The app provides technical means for achieving the desired tooth-brushing behavior, i.e., it incorporates a brushing timer. This provides practical support with brushing according to the 5-step method, showing each step during brushing.
	Adolescents receive cues to tooth-brushing (cues to action, habit formation).	- Prompts/cues: i.e., introducing or defining environmental or social stimulus with the purpose of prompting or cueing the behavior. The prompt or cue would normally occur at the time or place of performance (BCT 7.1) [2].	Cues work best when people are allowed to select and provide their own cues.	The app has an option for setting reminders, which are sent as push-notifications that function as "cues to action". The adolescents will be reminded to brush their teeth at a time that fits in with their daily routine.
PO5	Adolescents evaluate their tooth-brushing behavior, their dental plaque levels, and the effect of brushing on these levels.			
	Change objectives (determinant)	Theoretical methods/BCTs	Theoretical requirements	Practical strategies
	Adolescents monitor their tooth-brushing behavior and dental plaque levels (awareness, self-regulatory skills/action control).	- Self-monitoring of the behavior (BCT 2.3): i.e., the person monitors and records their behavior as part of a behavior change strategy [2].<br><br>- Self-monitoring of the outcome of behavior (BCT 2.3): i.e., the person monitors and records the outcome of their behavior as part of a behavior change strategy [2].	Commitment and motivation are required to use the self-monitoring part of the app.<br><br>The data must be interpreted and used.	Upon installing the app in the orthodontic clinic, a dental hygienist briefly shows the adolescent how to monitor their tooth-brushing and dental plaque levels.<br><br>Adolescents fill out their tooth-brushing frequency in the app every day. If they fail to complete the monitoring, a prompt message is sent the next day.
	Adolescents examine how well their performance	- Reviewing behavior goal(s) (BCT 1.5): i.e., reviewing behavior	Requires awareness of the oral health	Based on the information obtained from the brushing timer and the self-monitoring records,

	corresponds to agreed tooth-brushing goals, and consider modifying goals accordingly (skills and goal pursuit).	goal(s) jointly with the person and considering modifying goal(s) or behavior change strategy in the light of achievement. This may lead to re-setting the same goal, to a small change in that goal, or to setting a new goal rather than (or in addition to) the first; or to no change [2].<br><br>Discrepancy between the current behavior and goal (BCT 1.6): i.e., drawing attention to discrepancies between a person's current behavior (in term of the form, frequency, duration, and intensity of that behavior) and the person's previously set outcome goals or action plans (goes beyond self-monitoring of behavior).	recommendations.	the app provides personal feedback once a week on whether the person's performance corresponds to the agreed goals and asks them to consider modifying goals accordingly.
	Adolescents monitor their dental plaque levels (awareness, self-regulatory skills) and compare it with goal (awareness, self-regulatory skills).	- Using disclosing tablets to visualize and evaluate dental plaque [2].<br><br>- Prompting self-monitoring of the outcome of the behavior (BCT 2.4): i.e., establishing a method for the person to monitor and record the outcome(s) of their behavior as part of a behavior-change strategy [2].<br><br>- Providing feedback on the outcome of behavior (BCT 2.7): i.e., monitoring and providing feedback on the outcome of the behavior [1, 2].	Adolescents need to possess the sub-skill: identifying which dental surfaces are clean and which are covered with dental plaque.	Once a week the app asks adolescents to use disclosing tablets to visualize the dental plaque and to take a selfie of the results (this is the same procedure as during registration). It then asks them to designate the position of the dental plaque on the selfie by clicking on the screen. Based on the number of clicks, the app compares the number of clicks (plaque levels) with the results of the previous week, and provides feedback accordingly.
PO6	If they have difficulty attaining their tooth-brushing/dental plaque goal, adolescents identify possible solutions.			
	Change objectives (determinant)	Theoretical methods/BCTs	Theoretical requirements	Practical strategies
	Adolescents identify and anticipate barriers and ways to overcome them (coping planning, action control)	- Problem solving/prompt barrier identification (BCT 1.2): i.e., analyzing, or prompting the person to analyze, factors influencing the behavior; and	Barrier identification without solutions is not sufficient.<br><br>Requires identification of the barriers and	If a person's goal has not been achieved, volitional help sheets will be presented to help them identify barriers preventing them from proper tooth brushing and their possible solutions. The results of this volitional help sheets are presented as their

	Adolescents gain confidence to deal with possible barriers (coping self-efficacy).	generating or selecting strategies that include overcoming barriers and/or facilitators (includes "relapse prevention" and "coping planning"[[1, 2].<br><br>- Setting implementation intentions: i.e., prompting the making of if-then plans that link situational cues with responses that are effective in attaining goals or desired outcomes [1].	possible solutions/coping responses.	coping plan, which will be stored at the main page of the app. This coping plan is formulated as an implementation intention ("If difficult situation X arises, then I'll do Y").
	Adolescents enlist others to help overcome barriers (social influences).	- Mobilizing social support: i.e., advising on, arranging, or providing social support for performance of the behavior [1].	Adolescents are motivated to ask significant others.	If needed, adolescents are guided in asking significant others to support tooth brushing. When necessary, the app suggests asking parents and/or orthodontists for support, e.g., helping or demonstrating how to brush teeth.
PO7	Adolescents maintain the desired tooth-brushing behavior.			
	Change objectives (determinant)	Theoretical methods/BCTs	Theoretical requirements	Practical strategies
	Adolescents gain confidence in maintaining tooth-brushing behavior (maintenance self-efficacy).<br><br>Adolescents feel positive about tooth-brushing (outcome-expectancies).<br><br>Adolescents believe that long-term benefits can be achieved by maintaining tooth brushing over time (attitude).	- Belief selection: i.e., using messages designed to strengthen positive beliefs, weaken negative beliefs, and introduce new beliefs [1].<br><br>- Framing: Using gain-framed messages emphasizing the advantages of performing the healthy behavior[1].	Before choosing the beliefs on which to intervene, the individual's current attitudinal, normative, and efficacy beliefs should be investigated. Gain-framed messages are more readily accepted and prevent defensive reactions.	The app provides positive personal text-messages about favorable outcomes and the benefits of tooth brushing, (i.e., personal motives which are asked by the app when starting the app).

BCT: Behavior Change Technique; PO: Performance objectives.
* Many methods have clearly been defined, linked with theories of behavior change and classified into internationally recognized taxonomies of behavior-change techniques [1, 2]. These taxonomies were used to code the content of the program with standardized definitions of the behavior-change techniques (BCT's) classified by these taxonomies [2].

1. Kok G, Gottlieb NH, Peters GJ, Mullen PD, Parcel GS, Ruiter RA, Fernández ME, Markham C & Bartholomew LK. A taxonomy of behaviour change methods: an Intervention Mapping approach. Health Psychol Rev 2016 Sep;10(3):297-312. PMID:26262912
2. Michie S, Richardson M, Johnston M, Abraham C, Francis J, Hardeman W, Eccles MP, Cane J, Wood CE (2013) The Behavior Change Technique Taxonomy (v1) of 93 Hierarchically

Clustered Techniques: Builing an International Consensus for the Reporting of Behaviour Change Interventions. Ann Behave Med 2013 Aug;46(1):81-95. DOI:10.1007/s12160-013-9486-6

3. Boyd RL.Longitudinal evaluation of a system for self-monitoring plaque control effectiveness in orthodontic patients. J Clin Periodontol Jul;10(4),380-8. PMID:6577031
